# Seasonal variations in ventricular repolarization and tachyarrhythmias in hibernating brown bears (*Ursus arctos arctos*)

**DOI:** 10.14814/phy2.70531

**Published:** 2025-08-29

**Authors:** Lucas Alexander Lindberg, Boris Fuchs, Alina Lynn Evans, Timothy Laske, Anna Björkenheim, Ole Fröbert, Lisa Amalie Gottlieb

**Affiliations:** ^1^ Department of Biomedical Sciences University of Copenhagen Copenhagen Denmark; ^2^ Department of Forestry and Wildlife Management, Faculty of Applied Ecology and Biotechnology Inland Norway University of Applied Sciences Koppang Norway; ^3^ Medtronic Inc. Mounds View Minnesota USA; ^4^ University of Minnesota Minneapolis Minnesota USA; ^5^ Department of Cardiology, School of Medical Sciences Örebro University Örebro Sweden; ^6^ Department of Clinical Medicine, Faculty of Health Aarhus University Aarhus Denmark

**Keywords:** brown bear, electrocardiography, electrophysiology, hibernation, QT interval, ventricular tachyarrhythmias

## Abstract

In humans, hypothermia prolongs ventricular repolarization and associates with sustained ventricular tachyarrhythmias. In bears, body temperature drops during hibernation similar to moderate human hypothermia, yet they rarely face fatal outcomes during the winter. This suggests protective adaptations in bear electrophysiology. We studied seasonality in ursine ventricular repolarization by analyzing >1 year electrocardiogram (ECG) recordings from loop recorders implanted in 57 free‐ranging Eurasian brown bears. In sinus rhythm, bears exhibited significantly longer RR, QT, and T_peak_‐T_end_ intervals (2441 ± 470, 508 ± 50, and 53 ± 8 ms, respectively) during hibernation than in the active period (649 ± 323, 232 ± 39, and 29 ± 5 ms, respectively). Optimal heart rate correction of QT interval (QT/RR^0.435^) demonstrated significant prolongation during hibernation. QT and T_peak_‐T_end_ intervals remained longer during hibernation than in the active period, even when comparing ECGs with similar RR intervals in the two periods. Ventricular fibrillation occurred in four bears shot during licensed hunting in summer, which led to death. In conclusion, seasonal variations in ventricular repolarization in bears appear, at least partially, independently of heart rate. Compared to humans, ventricular repolarization is slower but more homogeneous. These findings, combined with the absence of fatalities during hibernation, support the theory of protective electrophysiological adaptations in bears. Insights into the underlying mechanisms have biomimetic potential for human therapy.

## 1 INTRODUCTION

Accidental hypothermia is a frequent cause of death worldwide, especially in colder climates and in mass casualty events such as avalanches (Paal et al., 2022). In the Arctic region, the cold weather poses significant risks, especially to homeless individuals and those who work or engage in outdoor activities (Rolf & Gallagher, 2018). While targeted temperature management is used therapeutically following cardiac arrest and during cardiac surgery, hypothermia is also strongly associated with arrhythmias (Mattu et al., 2002). Moderate to severe drops in body temperature are known to induce arrhythmias, including atrial fibrillation and ventricular fibrillation (VF), and even minor decreases in ambient temperature have been linked to an increased risk of ventricular tachyarrhythmias (Mattu et al., 2002; McGuinn et al., 2013). In addition, hypothermia‐induced cardiac arrest is often refractory to defibrillation and thus, current clinical guidelines recommend delaying excessive attempts at defibrillation until the core body temperature has been rewarmed to >30°C (Lott et al., 2021).

**FIGURE 1:**
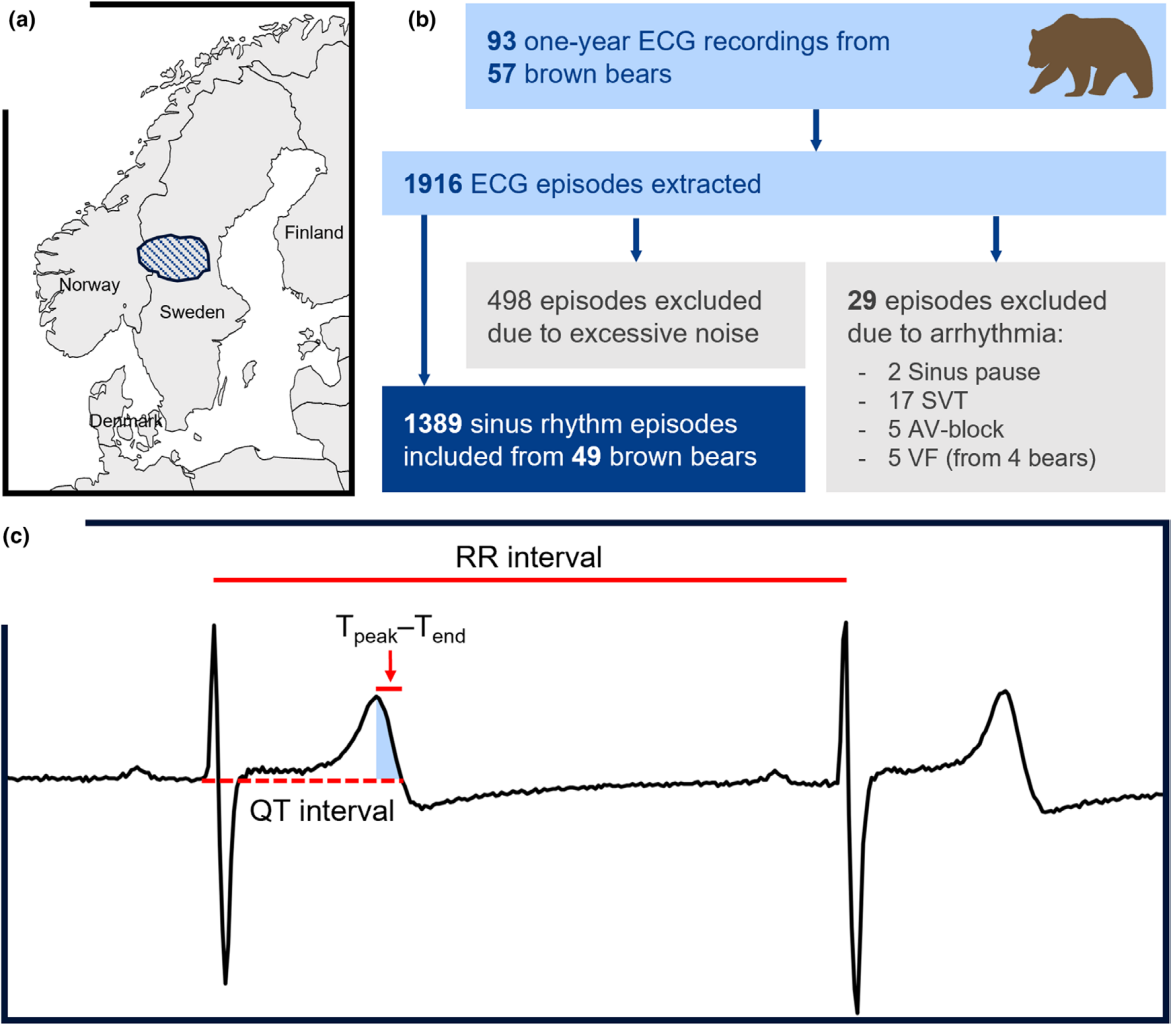
Screening and analysis of ECG recordings from free‐ranging brown bears. (a) Free‐ranging brown bears were recruited in south‐central Sweden. (b) Overview of the screening process for ECG recordings spanning from 1 to 3 years from 57 bears. Recordings were manually classified as sinus rhythm, noise, or arrhythmias. (c) Illustration of relevant ECG parameters used in the analysis. The RR interval is the inverse of heart rate, the QT interval indicates ventricular repolarization duration, and the T_peak_‐T_end_ interval reflects the ventricular repolarization heterogeneity. ECG, electrocardiogram; HR, heart rate; RR, R‐R interval; QT, QT interval; T_peak_‐T_end_, peak‐to‐end of T wave interval; SVT, supraventricular tachycardia; AV, atrioventricular; VF, ventricular fibrillation.

Ventricular tachyarrhythmias are linked to alterations in ventricular repolarization, typically characterized by slowed repolarization and/or enhanced dispersion of repolarizing currents (Boukens et al., 2016). The electrocardiogram (ECG) allows for non‐invasive assessment of ventricular repolarization, with key markers including the QT interval, T_peak_–T_end_ interval, and the T_peak_–T_end_/QT interval ratio (Figure 1). The QT interval on the ECG measures the time from the beginning of ventricular depolarization (Q wave) to the end of ventricular repolarization (T wave). Changes in the QT interval largely reflect changes in total repolarization time, as depolarization times vary only slightly. The interval from the peak to the end of the T wave (T_peak_–T_end_ interval) is likewise a marker of ventricular repolarization in humans. Initially, the T_peak_–T_end_ interval was thought to reflect the transmural gradient of repolarization in the ventricular wall; however, studies in explanted whole pig hearts have shown that it represents the overall repolarization gradient involving both ventricles (Meijborg et al., 2014; Opthof et al., 2007). Because heart rate (HR; the inverse of the interval from one R wave to the next, known as the RR interval on the ECG) influences ventricular repolarization time, the QT interval is often normalized to HR. Likewise, the T_peak_–T_end_/QT interval ratio eliminates the HR effect on repolarization (Zumhagen et al., 2016).

Therefore, these ECG markers can help identify pathologically prolonged and heterogeneous ventricular repolarization, which are known risk factors for fatal ventricular tachyarrhythmias in humans (Algra et al., 1991; Panikkath et al., 2011). In humans, the upper physiological limit of the HR‐corrected QT interval is set at 480 ms due to the higher risk of early afterdepolarizations, torsades de pointes, and VF in patients with a QT interval exceeding this value (Kass & Moss, 2003). A high T_peak_–T_end_/QT interval ratio may also be associated with sudden cardiac death in humans (Mugnai et al., 2017; Zumhagen et al., 2016). Moreover, Haïssaguerre et al. and others have identified early repolarization patterns on ECG, characterized by slurring or notching at the terminal portion of the QRS complex, as being associated with a heightened risk of VF (Haïssaguerre et al., 2008). In 1953, John Osborn described a similar phenomenon in hypothermic dogs, referring to it as “a secondary wave closely following the S‐wave, so closely that it appears to be part of the QRS complex” (Osborn, 1953). These so‐called Osborn waves have been shown to increase in amplitude as body temperature decreases and have been linked to hypothermia‐induced ventricular tachyarrhythmias in humans (Okada et al., 2020). In addition, prolongation of the QT interval is observed in humans with mild hypothermia (Mattu et al., 2002). Altogether, these findings suggest that the arrhythmogenic mechanisms associated with hypothermia may share common pathways with those leading to VF in normothermic individuals.

The Eurasian brown bear (*Ursus arctos arctos*) in Scandinavia hibernates for 5–6 months each year (Evans, Singh, Friebe, et al., 2016; Friebe et al., 2001). During the hibernation period, body temperature decreases by 4°C–5°C below normal levels, yet mortality remains rare (Friebe et al., 2014; Thienel et al., 2023). This suggests that brown bears possess protective adaptations in ventricular repolarization during hibernation that prevent hypothermia‐induced ventricular arrhythmias. The brown bear, therefore, offers a promising model for biomimetic therapies, aiming at preventing or treating ventricular tachyarrhythmias. In this study, we investigated seasonal variations in ventricular repolarization and arrhythmias in free‐ranging bears.

## 2 RESULTS

**FIGURE 2:**
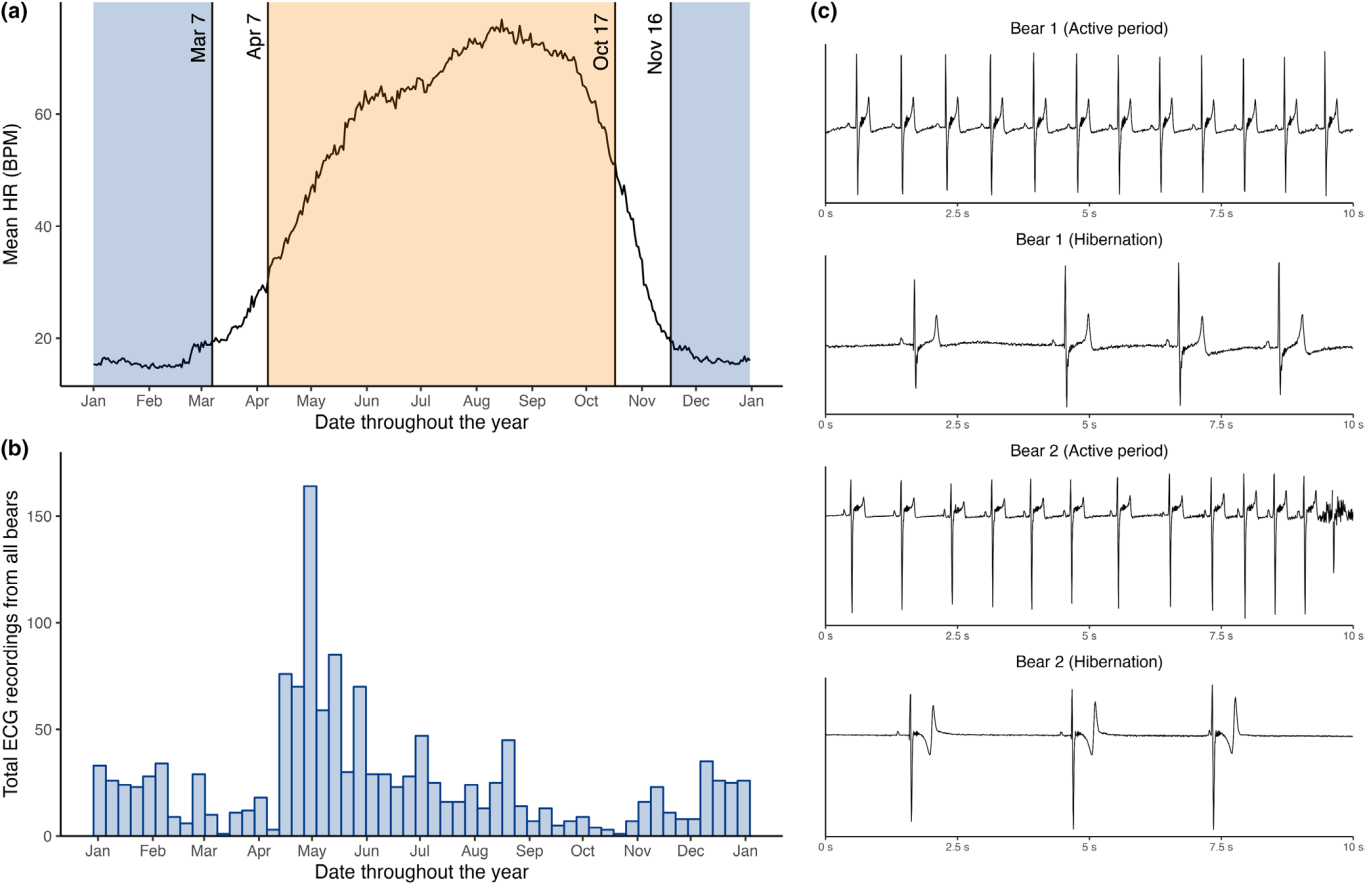
Annual heart rate variation and distribution of ECG recordings. (a) Division into the active period (orange), hibernation (blue), and two 30‐day transition periods is based on mean diurnal heart rate measurements (black lines) from brown bears (*Ursus arctos*) measured in Scandinavia between 2014 and 2020. (b) Histogram showing the distribution of recorded episodes throughout the year. (c) Representative ECGs showing sinus rhythm from the active period and hibernation in two brown bears (*Ursus arctos*) (bear 1: 27 August 2015 and 15 January 2016; bear 2: 11 June 2015 and 4 January 2015). Amplification ringing seen as multiple high‐frequency deflections after the S wave occurred after short QRS intervals. No Osborn waves were observed.

This study retrieved 93 one‐year ECG recordings from 57 Eurasian brown bears (27 females and 22 males) with a median age at study inclusion of 5 years (range 1–22 years) and body weight mean of 78 ± 57 (standard deviation [SD]) kg (Figure 1). The bears were captured between 2014 and 2020 in south‐central Sweden. In total, we retrieved 1916 ECG episodes from implantable loop recorder (ILR) devices. After manual screening, we included 1389 ECG tracings of sinus rhythm from 49 bears for further analysis, while 498 tracings with excessive noise were excluded. Figure 2 shows the time‐of‐year distribution of recorded sinus rhythm tracings. Most of the stored ECG episodes were recorded during late spring, coinciding with ILR implantation.

### 2.1 Seasonal electrocardiographic variation

Figure 2c depicts representative ECG tracings in sinus rhythm during the active period and hibernation in two bears. No Osborn waves occurred. In the majority of the ECGs, amplification ringing was observed after the S wave due to a short QRS interval.

**FIGURE 3:**
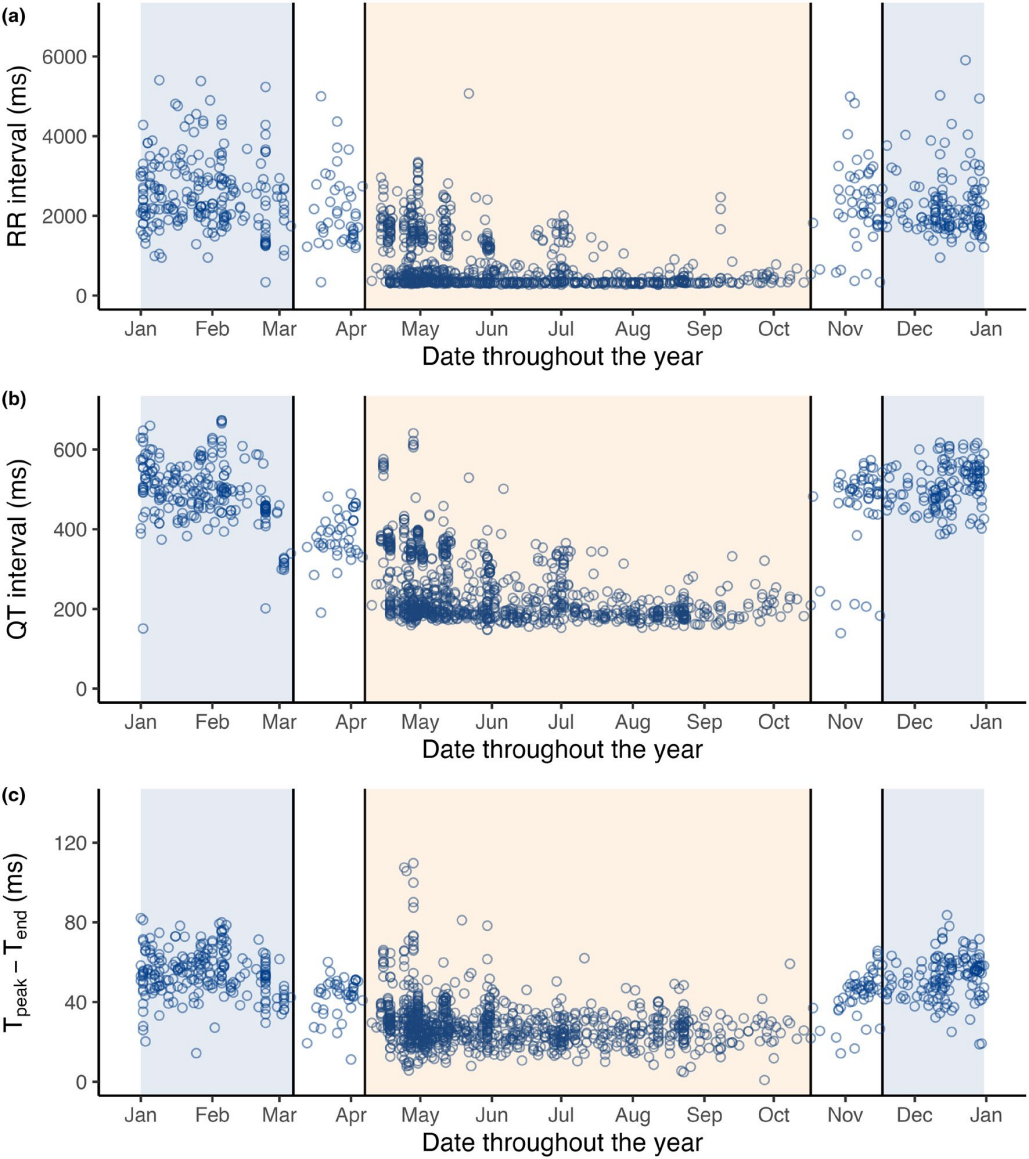
ECG parameters throughout the year. Scatterplots of ECG parameters throughout the year, where each point marks a representative beat from an ECG recording. (a) RR interval. (b) QT interval. (c) T_peak_–T_end_ interval.

Large variations in RR, QT, and T_peak_–T_end_ intervals occurred throughout the year, with the longest intervals during hibernation. These shortened in spring transition and reached their shortest intervals during the active season (Figure 3). Table 1 lists averaged ECG parameters for the four seasonal periods. The RR interval shortened by 72% from hibernation to the active period, while the QT and T_peak_–T_end_ intervals shortened by 54% and 45%, respectively. The QRS interval was also longer during hibernation, though its shortening in summer was less profound, with only a 5% change.

**TABLE 1:** Seasonal variation in electrocardiographic parameters in sinus rhythm from free‐ranging brown bears (*Ursus arctos*) in Scandinavia.

	Hibernation (H)	Spring transition (TrS)	Active period (A)	Autumn transition (TrA)	All‐season, *p*‐value	Pairwise season, *p*‐value
Episodes, n	360	43	937	49	NA	NA
Bears, *n*	32	7	49	10	NA	NA
RR interval (ms) mean ± SD	2441 ± 470	1785 ± 782	649 ± 323	1704 ± 1035	<0.0001	H vs. TrS = 0.004; H vs. A<0.00001; H vs. TrA = 0.214; TrS vs. A<0.00001; TrS vs. TrA = 0.509; A vs. TrA<0.00001
QRS interval (ms)	63 ± 8	58 ± 6	60 ± 5	62 ± 8	<0.0001	H vs. TrS<0.00001; H vs. A<0.00001; H vs. TrA = 0.899; TrS vs. A <0.104; TrS vs. TrA = 0.0002; A vs. TrA = 0.002
QT interval (ms) mean ± SD	508 ± 50	393 ± 103	232 ± 39	390 ± 147	<0.0001	H vs. TrS<0.00001; H vs. A<0.00001; H vs. TrA = 0.0252; TrS vs. A<0.00001; TrS vs. TrA = 0.0006; A vs. TrA<0.00001
T_peak_–T_end_ (ms) mean ± SD	53 ± 8	43 ± 11	29 ± 5	38 ± 14	<0.0001	H vs. TrS<0.00001; H vs. A<0.00001; H vs. TrA<0.0001; TrS vs. A<0.00001; TrS vs. TrA = 0.789; A vs. TrA<0.00001
T_peak_–T_end_/QT ratio mean ± SD	0.103 ± 0.013	0.113 ± 0.020	0.127 ± 0.020	0.099 ± 0.017	<0.0001	H vs. TrS = 0.9998; H vs. A <0.00001; H vs. TrA = 0.2452; TrS vs. A = 0.0758; TrS vs. TrA = 0.6014; A vs. TrA = 0.00006
QTc_P_ (ms) mean ± SD	355 ± 41	324 ± 62	303 ± 20	321 ± 54	<0.0001	H vs. TrS = 0.00017; H vs. A<0.00001; H vs. TrA = 0.8220; TrS vs. A = 0.7308; TrS vs. TrA = 0.0301; A vs. TrA<0.00001

To assess whether ECG parameters indicated a warming vs. cooling effect, we compared the spring transition (transitioning from hypothermia to normothermia) to the autumn transition (transitioning from normothermia to hypothermia). While RR and T_peak_–T_end_ intervals were similar in both transition phases, QT intervals were longer and QRS intervals were shorter in spring transition compared to autumn transition (average differences: 3 ms, *p* = 0.0006, and 2 ms, *p* = 0.0002, respectively), indicating diverse thermal effects on ventricular ion channels.

### 2.2 Heart rate correction of ventricular repolarization in brown bears

**FIGURE 4:**
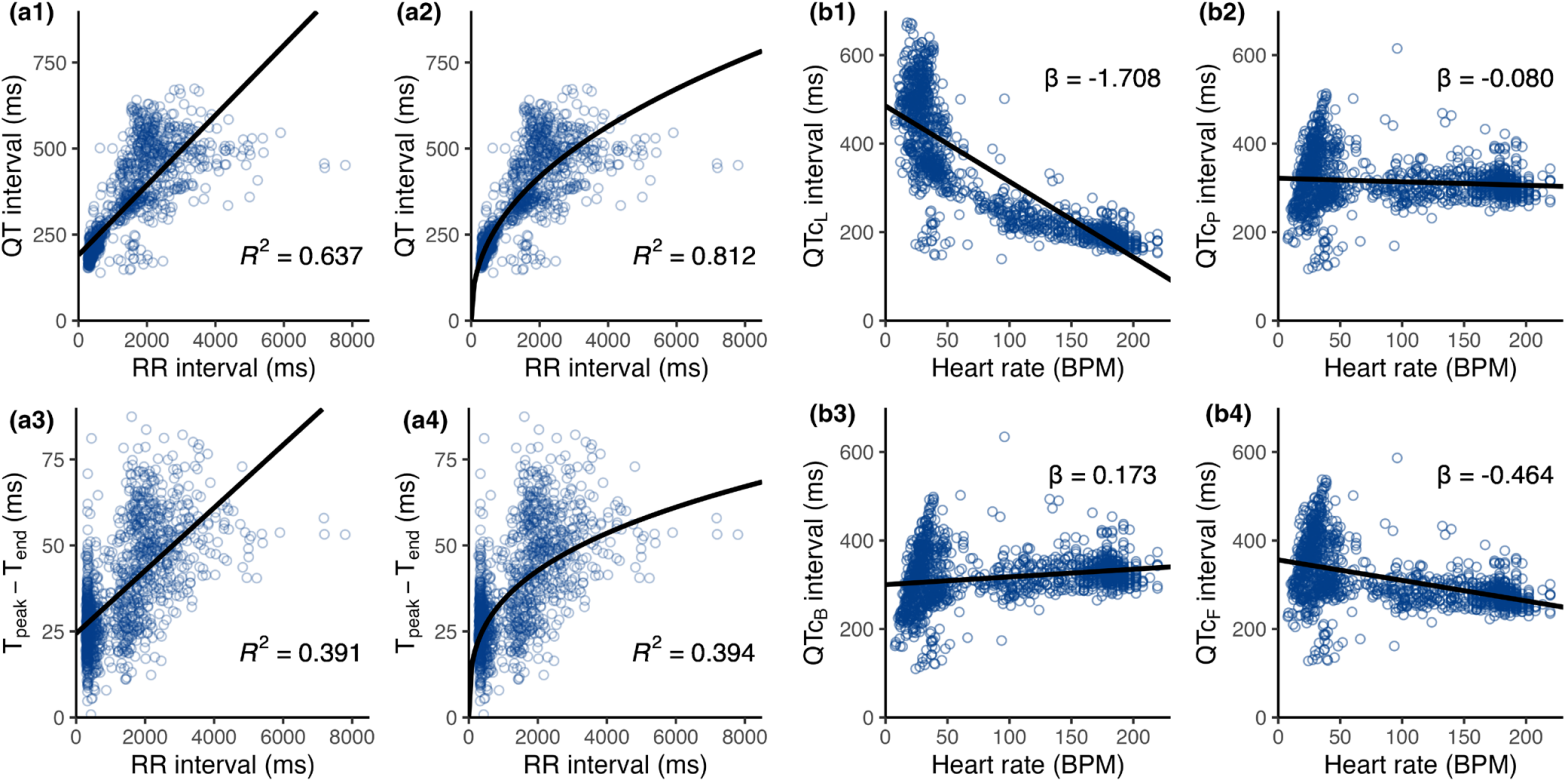
Heart‐rate dependency and correction of ECG parameters. (a) Scatterplots for QT and T_peak_–T_end_ intervals by RR interval from brown bears (*Ursus arctos*) measured in Scandinavia between 2014 and 2020. Panels a1 and a3 show linear regressions, while a2 and a4 display power regressions. (b) Scatterplots for the HR‐corrected QT intervals by HR with the linear regression slope (*β*) indicated. Panel b1 shows the linear correction, b2 the power correction, b3 Bazett's formula, and b4 Fridericia's formula.

Since ventricular repolarization depends on HR, methods for HR correction of QT and T_peak_‐T_end_ intervals were deployed to evaluate truly intrinsic, seasonal changes in ventricular repolarization. The linear regression showed a moderate correlation between QT and RR interval, while the power regressions demonstrated the strongest correlation. This corresponds to a correction formula of ${\mathrm{QTc}}_{P}=\mathrm{QT}/{\mathrm{RR}}^{0.435}$, with the RR exponent lying between the exponents in Bazett's and Fridericia's formulae (Figure 4a, Table 2). Meanwhile, the T_peak_–T_end_ interval only weakly correlated with the RR interval in both linear and power regressions (Figure 4a).

**TABLE 2:** Calculated formulae and their slopes for heart rate correction of QT interval.

Method	Formula	Slope (β)	*R* ^2^
Linear correction	${\mathrm{QTc}}_{L}=\mathrm{QT}+0.102\left(1-\mathrm{RR}\right)$	−1.708	0.721
Power correction	${\mathrm{QTc}}_{P}=\mathrm{QT}/{\mathrm{RR}}^{0.435}$	−0.080	0.009
Bazett's method	${\mathrm{QTc}}_{B}=\mathrm{QT}/{\mathrm{RR}}^{1/2}$	0.173	0.045
Fridericia's method	${\mathrm{QTc}}_{F}=\mathrm{QT}/{\mathrm{RR}}^{1/3}$	−0.464	0.240

Comparing our calculated linear and power corrections of QT intervals to Bazett's and Fridericia's corrections, we observed the flattest slope (indicating optimal HR correction) for our power correction ($\beta_{P}=-0.080$), followed by Bazett's method ($\beta_{B}=0.173$; Figure 4b, Table 2).

### 2.3 Seasonal variation in the relation between heart rate and ventricular repolarization

**FIGURE 5:**
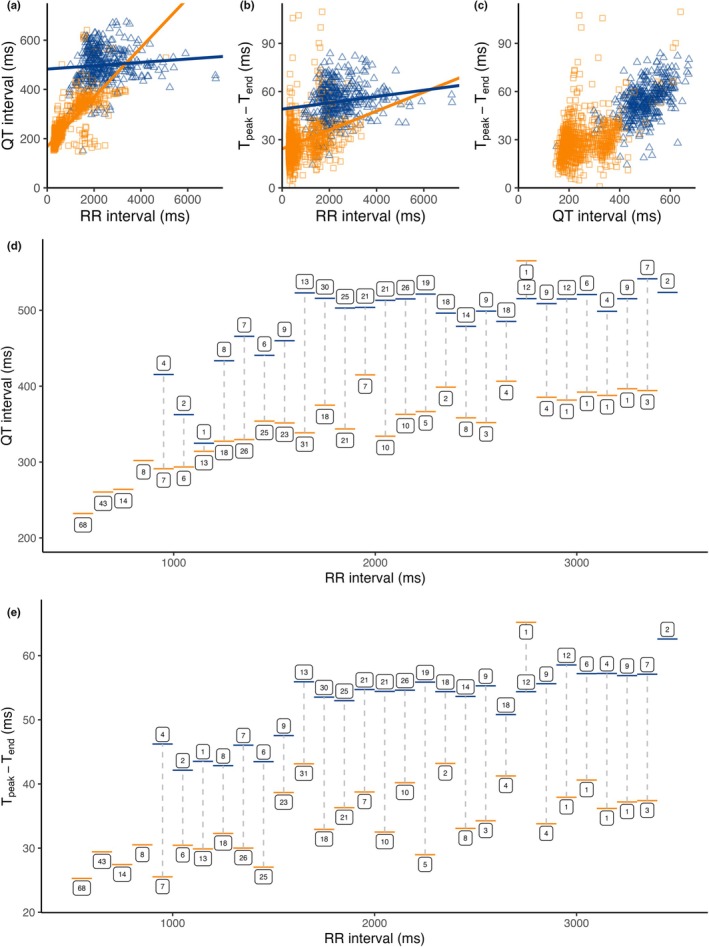
Seasonal variation in ventricular repolarization. (a) Scatterplot with linear regression lines for QT interval in relation to RR intervals from Eurasian brown bears (*Ursus arctos*), divided into the active period (orange squares) and hibernation (blue triangles); transition periods were omitted. (b) Likewise, T_peak_–T_end_ by RR interval and (c) T_peak_–T_end_ by QT interval. (d) QT interval and (e) T_peak_–T_end_ interval of beats with RR intervals grouped in 100‐ms segments, showing longer ventricular repolarization duration and dispersion during hibernation (blue) despite similar RR intervals, compared to the active period (orange). Horizontal lines represent the RR interval span (*X*‐axis) and the mean of the QT or T_peak_–T_end_ intervals (*Y*‐axis), with the boxed number indicating the number of ECG episodes per segment. Segments of RR intervals that include episodes from both the active period and hibernation are connected with a vertical, dotted line.

Figure 5 shows the QT/RR, T_peak_–T_end_/RR, and T_peak_–T_end_/QT interval data clustered into hibernation and the active period. The linear mixed‐effect model between RR interval and QT showed a significant interaction with season (*p* < 0.0001), indicating that HR dependency of ventricular repolarization changed with season. During the active season, a 100 ms increase in RR interval led to a 9.7 ms increase in QT interval, while a similar change in RR interval caused only a 1.1 ms increase in QT interval during hibernation.

Likewise, a seasonal difference between RR and T_peak_–T_end_ interval was found (p < 0.0001). Nevertheless, a 100 ms prolongation of the RR interval led to only a 0.6 ms increase in the T_peak_–T_end_ interval during the active period and 0.3 ms during hibernation.

### 2.4 Seasonal variation in ventricular repolarization independent of heart rate

The averaged T_peak_–T_end_/QT ratio was lower in hibernation compared to the active period (Table 1), but the linear regression between T_peak_–T_end_ and QT interval did not differ significantly between these two seasons (interaction *p* = 0.116; Figure 5c).

Moreover, QTc_P_ intervals were 15% longer in hibernation compared to the active period (Table 1), suggesting seasonal variation in ventricular repolarization independent of HR. To further explore this variation, we differentiated sinus rhythm beats into groups with similar RR intervals and observed that both QT and T_peak_–T_end_ intervals were longer during hibernation compared to the active period, despite having similar RR intervals (Figure 5d,e).

### 2.5 Ventricular tachyarrhythmias

**FIGURE 6:**
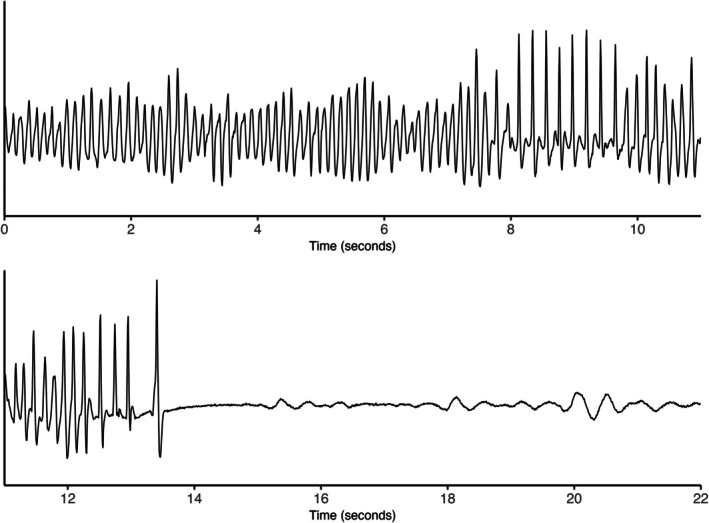
Fatal ventricular fibrillation in a brown bear. Tracing of a fatal VF episode in a male brown bear (*Ursus arctos*), recorded on 29 August 2014 at 04:51 UTC in central Sweden. The bear was shot during licensed hunting. This episode was the last recorded by the ILR; no subsequent activity or HR measurements were available. The onset of the arrhythmia was not stored.

In four bears, we identified VF, all of which were followed by death, being documented by a continuously averaged HR of 0. Figure 6 shows a representative tracing of a VF episode transitioning into asystole, though the tracing does not capture the precise onset of the arrhythmia. VF occurred immediately after the bear was legally shot. We did not observe any episodes of non‐sustained ventricular tachyarrhythmias. The remaining arrhythmias were identified as sinus pauses (2 tracings from 1 bear), atrioventricular block (5 tracings from 4 bears), and supraventricular tachyarrhythmias (17 tracings from 13 bears).

## 3 DISCUSSION

In this descriptive study of long‐term ECG monitoring in free‐ranging Eurasian brown bears, we observed a hibernation‐related prolongation in ventricular repolarization that was independent of the slower HR and not likely associated with ventricular tachyarrhythmias. Indeed, VF only occurred in the active season after gunshot, leading to death. The arrhythmias were, therefore, not related to hypothermia or other physiological phenomena.

### 3.1 Seasonal variation in heart rate and repolarization

In free‐ranging brown bears, we observed a large drop in HR during hibernation compared to all other seasons, while QT and T_peak_–T_end_ intervals were markedly prolonged. Others have similarly reported extensive HR slowing in hibernating brown and grizzly bears compared to the active period (Evans et al., 2023; Nelson et al., 2003). The results of the statistical mixed‐effect model indicated that the HR dependency of ventricular repolarization varies with season (Figure 5a,b). Moreover, the seasonal difference in QT and T_peak_–T_end_ interval appeared to depend not only on HR but also on intrinsic seasonal changes in repolarization independent of HR (Figure 5d).

Few studies have investigated seasonal variation in ventricular repolarization in bears. Folk and colleagues measured lower HR and longer QT interval in hibernation compared to during sleep in the active period in two free‐ranging Alaskan brown bears (HR 19 ± 7 vs. 46 ± 2 beats per minute [BPM] and QT 474 ± 111 vs. 226 ± 57 ms, hibernation vs. active period, respectively) (Folk et al., 2008). Gandolf et al. reported a QT interval of 253 ± 25 ms and a QTc using Bazett's formula of 273 ± 22 ms during the month of April in 22 brown bears anesthetized with tiletamine, zolazepam, and medetomidine (Gandolf et al., 2010). These values closely resemble our observations in active summer, suggesting that the anesthetic agents likely had a minimal impact on cardiac electrophysiology.

### 3.2 Physiological explanations for seasonal changes in repolarization

Hypothermia is known to prolong ventricular repolarization in humans, both accidental hypothermia and during therapeutic neuroprotection after cardiac arrest resuscitation (Khan et al., 2010; Mattu et al., 2002). The brown bear experiences temperature drops of 4°C–5°C during hibernation (Friebe et al., 2014; Thienel et al., 2023), which are similar to mild to moderate hypothermia in humans. Hence, the alterations in repolarization in the hibernating brown bear may be directly related to hypothermia, and the bear can serve as a more suitable model for studying hypothermia in humans compared to smaller hibernating mammals, whose torpor states are far more extreme, with body temperatures approaching the freezing point (Maistrovski et al., 2012).

Dietrichs et al. showed that moderate hypothermia to 31°C prolongs the QT interval by 50% in isolated rabbit hearts (Dietrichs et al., 2020). The study found that the repolarization time and action potential duration increased at 31°C, and the VF threshold estimated by burst‐pacing induction was significantly reduced, indicating a higher susceptibility to arrhythmia. This is likely due to alterations in ion channel and gap junction properties, resulting in temperature‐dependent changes in repolarization and conduction. Indeed, Fedorov and colleagues demonstrated upregulation of connexin proteins and faster conduction velocity in the papillary muscle of Siberian ground squirrels during hibernation compared to the active period (Fedorov et al., 2005). However, when hibernating, these animals have a core body temperature as low as –2°C without freezing (Fedorov et al., 2005), thereby differing from Eurasian brown bears that maintain a body temperature >30°C.

The autonomic nervous system (ANS) regulates HR and influences cardiac electrophysiology through autonomic nerve terminals in the sinus node and myocardium. A strong parasympathetic response is believed to occur in bears during hibernation, as shown by previous observations of frequent sinus arrhythmias, sinus bradycardia, and sinus pauses (Gottlieb et al., 2023; Støen et al., 2015). Moreover, the ANS directly alters ventricular repolarization. Meijborg et al. reported that left stellate ganglion stimulation caused a biphasic response—with an initial prolongation followed by a shortening of ventricular repolarization time in living pigs (Meijborg et al., 2020). This could explain the shortened QT intervals in the bears during summer, coinciding with higher sympathetic levels.

HR variability is indicative of ANS activity, and a previous study in the Eurasian brown bear population showed a sharp decrease in HR variability (measured as the standard deviation of the average NN intervals) once the bears entered their dens to hibernate. The decrease was suspected to be caused by enhanced parasympathetic and/or reduced sympathetic activity (Evans, Singh, Friebe, et al., 2016). Altogether, these findings suggest a potential link between seasonal ANS regulation and the observed seasonal changes in ventricular repolarization in free‐ranging bears.

Cardiac tissue composition has previously been shown to alter during hibernation in bears. Nelson et al. reported an upregulation of a stiffer isoform of titin, along with downregulation of a more elastic titin isoform, in left ventricular tissue samples from grizzly bears during hibernation compared to the active period (Nelson et al., 2008). Despite slower HR, left ventricle dilation during diastole, as measured by echocardiography, did not exhibit seasonal variation, which may be due to the titin isoform change. However, it remains unclear whether myocardial wall tension is altered in a way that modulates the mechano‐electrical coupling, ultimately influencing ventricular electrophysiology and potentially contributing to arrhythmia formation (Quinn & Kohl, 2021). Nevertheless, the cellular and molecular mechanisms underlying cardiac electrophysiology in hibernating bears remain poorly understood.

### 3.3 Ventricular repolarization

The bears showed remarkable seasonal changes in markers of ventricular repolarization without evident risk for ventricular tachyarrhythmias. In humans, HR‐corrected QT interval above 480 ms constitutes a risk factor for afterdepolarizations, torsades de pointes, and VF in patients (Kass & Moss, 2003). Our data (Figure 4b) showed that free‐ranging bears exceeded these HR‐corrected QT interval values without any pathological outcome.

The T_peak_–T_end_ interval represents the overall repolarization gradient involving both ventricles (Meijborg et al., 2014; Opthof et al., 2007). The T_peak_–T_end_ interval values in the bears were shorter than those in healthy humans, averaging 91 ± 13 (SD) ms (Li et al., 2022), indicating that bears naturally exhibit homogenous ventricular repolarization. Moreover, the T_peak_–T_end_ /QT interval ratio is reported to be higher (0.24) in patient with Brugada syndrome and a high risk of malignant arrhythmias compared to patients with low risk (0.19), while others do not observe such differences (Mugnai et al., 2017; Zumhagen et al., 2016). Nevertheless in the bears, however, the T_peak_–T_end_/QT interval ratio (ranging 0.10–0.13 throughout the year) was markedly lower than values observed in healthy humans without risk of cardiac morbidity (average 0.22 ± 0.06 SD) (Li et al., 2022).

Insights into how bears achieve low heterogeneity in ventricular repolarization and remain protected from ventricular tachyarrhythmias despite remarkably long repolarization times could lead to biomimetic approaches for improving the treatment of idiopathic and hypothermia‐related ventricular tachyarrhythmia in humans.

### 3.4 Heart rate dependent repolarization and QT interval correction

The power regression of QT to RR intervals showed a flatter slope—and thus a better HR‐correction compared to Bazett's and Fridericia's methods in bears. To our knowledge, no other correction formulae have been calculated for brown bears. The adaptability of QT intervals varies between species, and QT interval correction must take species variation into account (Boukens et al., 2014). Previous studies have found Bazett's method to be optimal for crab‐eating macaques (*Macaca fascicularis*), while van de Water's formula (${\mathrm{QT}}_{C}=\mathrm{QT}-0.087\left(\mathrm{RR}-1000\right)$) has proven superior in anesthetized beagle dogs (Soloviev et al., 2006; Van de Water et al., 1989).

### 3.5 Limitations

Our study subjects were free‐ranging bears in their natural habitats. While this allows for the observation of real‐life physiology with minimal anesthesia, it also limits our ability to conduct experimental studies. The capturing and immobilization of the bear is a considerable disturbance that has physiological implications, including changes in HR and body temperature, which require time to return to baseline (Evans, Singh, Fuchs, et al., 2016).

Although the BearWare software provided important data and improved the efficacy of the ILR, undersensing of the ECG and oversensing of electrical noise and/or T waves resulted in some erroneous recordings. Due to noise and the low sampling rate of the ECG recordings, we were unable to include PR interval data, as the excessive noise made it impossible to reliably determine the onset of the P wave.

Most of the stored ECG episodes occurred during late spring, coinciding with the period when the ILRs were implanted. The extremes of the ursine heart triggered the ILR far more frequently than in humans, causing the number of events to exceed the device's storage capacity. As a result, we cannot determine the true incidence rate of arrhythmia, nor can we conclude the absence of arrhythmia in hibernating brown bears based solely on our data.

### 3.6 Conclusion

Brown bears experience significant HR independent changes in ventricular repolarization during hibernation, as evidenced by prolonged QT and T_peak_–T_end_ intervals. This seasonal variation in ursine electrophysiology may serve as a foundation for cellular and molecular studies of the brown bear heart, offering a potential biomimetic approach for the management and treatment of arrhythmias in humans.

## 4 MATERIALS AND METHODS

As a part of the Scandinavian Brown Bear Project (www.brownbearproject.com), this study was conducted on free‐ranging, healthy brown bears and was ethically approved by the Swedish Ethical Committee on Animal Experiments (Application Numbers C47/9, C7/12, C18/15, C212/9, and C268/12) and the Swedish Environmental Protection Agency.

Field staff immobilized Eurasian brown bears (*Ursus arctos arctos*) in south‐central Sweden by darting them with sedatives from a helicopter during the summer season, as previously described (Arnemo & Evans, 2017). Body weight and sex were recorded. Each bear was fitted with a global positioning system (GPS) collar (Vectronic Aerospace GmbH, Berlin, Germany) and an ILR (Reveal XT, Medtronic Inc., Minneapolis, Minnesota, USA), placed subcutaneously next to the sternum.

### 4.1 ECG recording and extraction

The ILR recorded a single‐lead ECG before, during, and after arrhythmic events until the device memory was full. The ECG recordings had a sampling rate of 128 Hz. Custom software adapted for bears was used for arrhythmia detection and storage (BearWare, Medtronic Inc., Minneapolis, MN, USA) (Laske et al., 2018). The devices were programmed to identify and record arrhythmic events associated with heart rates of >176 BPM and pauses >4.5 s. These recorded episodes enable detailed evaluation of the associated ECGs.

We extracted all ECG recordings from the ILR. In many cases, the arrhythmia detection by BearWare software was faulty due to oversensing of myograms or motion artifact and/or undersensing of low amplitude signals during activity, and the ECG tracing showed normal sinus rhythm or excessive noise. Each episode was manually annotated based on overall signal quality, and episodes with excessive noise, that per definition did not visualize a single sinus/arrhythmic beat, were excluded since further analysis was not possible. Each episode was also evaluated based on its rhythm and ECG patterns to exclude arrhythmic episodes from the sinus rhythm analysis.

In addition, averaged HR in two‐minute intervals and the number of minutes per 15‐minute interval in which the bear was physically active were stored until the device was re‐interrogated or retrieved (Laske et al., 2018). Data were retrieved at recapture events or from bears killed during the license hunt, starting August 21 each year and lasting until quotas are filled.

### 4.2 ECG analysis

Conventional ECG analysis was performed on sinus rhythm tracings using a modified Pan–Tompkins' algorithm to detect QRS complexes (Pan & Tompkins, 1985). Based on QRS complex detections, a custom algorithm using local maxima and minima was applied to detect the Q, R, S, and T waves. The end of the T wave (T_end_) was determined using the tangent method, calculating the intersection between the major descending slope of the T wave and the isoelectric line. These methods enabled the calculation of QRS complex width and RR, QT, and T_peak_–T_end_ intervals.

Linear and power regression models were used to explore the HR dependency of QT and T_peak_–T_end_ intervals. Regression constants were used to devise a novel correction formula for corrected QT (QTc) intervals in brown bears, which was then compared to traditional HR correction methods, including Bazett's (${\mathrm{QTc}}_{B}=\mathrm{QT}/{\mathrm{RR}}^{1/2}$) and Fridericia's formula (${\mathrm{QTc}}_{F}=\mathrm{QT}/{\mathrm{RR}}^{1/3}$).

To further analyze the HR dependency on ventricular repolarization markers, ECG tracings were grouped by narrow RR intervals of 100 ms, and ventricular repolarization parameters were compared within these intervals across seasons.

### 4.3 Seasonal classification

ECG episodes were classified by season using HR data. Since the ILR did not record sufficient HR data in all bears, a mean HR for each day throughout a year using data from all years was calculated (Figure 1a). Based on these averaged data, four seasonal periods were defined: the active period, transition to hibernation during autumn, hibernation, and transition to the active period during spring. Hibernation onset was defined as the first day of the lowest overall HR + 5 BPM, occurring between September and December. The end of hibernation was determined as the last occurrence of the lowest overall HR + 5 BPM, occurring between February and July. The autumn transition period was defined as the 30 days preceding hibernation onset, while spring transition was defined as the 30 days following hibernation termination. The active period was defined as the time from the end of spring transition to the beginning of autumn transition.

For each bear, the average of each ECG parameter per seasonal period was calculated using all sinus rhythm tracings recorded during that period. All ECG analyses were performed using R Statistical Software (R Core Team, Vienna, Austria) with the *gsignal* and *zoo* packages (van Boxtel et al., 2021; Zeileis & Grothendieck, 2005).

### 4.4 Statistical analysis

In each sinus rhythm recording, one heartbeat with the median RR interval served as the representative beat for the entire tracing, and these measured ECG parameters were used for further statistical analysis. To account for the unbalanced dataset, we applied a linear mixed‐effect model of compound symmetry to the one‐beat data, regarding season as a fixed effect and each bear and ECG recording as random effects. If the multivariate model showed *p* < 0.05, a univariate Wald test with Tukey's method for multiple comparisons was performed to evaluate statistical differences between each season. To assess seasonal variation in the dependency between two ECG parameters (e.g., QT and RR), an interaction term was added to a linear regression analysis.

Finally, ECG data from each season were averaged for each bear, generating an overall mean ± standard deviation (SD) for all bears. All *p*‐values are reported. Statistical analyses were performed using R Statistical Software (R Core Team, Vienna, Austria) with the *LMMstar* and *multcomp* packages (Hothorn et al., 2008; Ozenne & Forman, 2024).

## AUTHOR CONTRIBUTIONS

Lucas Alexander Lindberg, Anna Björkenheim, Ole Fröbert, and Lisa Amalie Gottlieb designed the study. Lucas Alexander Lindberg and Lisa Amalie Gottlieb analyzed the data and prepared the first draft of the manuscript. Boris Fuchs, Timothy Laske, and Alina Lynn Evans performed the experiments. All authors contributed to the interpretation of the data and the manuscript writing.

## CONFLICT OF INTEREST STATEMENT

AB reports receiving speaker honoraria from Medtronic. TL is an employee of Medtronic.

LAG acts as an external reviewer for the Novartis Research Foundation.

## ETHICS STATEMENT

This study was conducted on free‐ranging, healthy brown bears and was ethically approved by the Swedish Ethical Committee on Animal Experiments (Application Numbers C47/9, C7/12, C18/15, C212/9, and C268/12) and the Swedish Environmental Protection Agency.

## Data Availability

The data that support the findings of this study are available on request from the corresponding author. The data are not publicly available due to privacy or ethical restrictions.
